# WHO guidance grounded in a comprehensive approach to sexual and reproductive health and human rights: topical pre-exposure prophylaxis

**DOI:** 10.7448/IAS.17.3.19279

**Published:** 2014-09-08

**Authors:** Manjula Lusti-Narasimhan, Rajat Khosla, Rachel Baggaley, Marleen Temmerman, Elizabeth McGrory, Tim Farley

**Affiliations:** 1 Department of Reproductive Health and Research, World Health Organization, Geneva, Switzerland; 2 Department of HIV/AIDS, World Health Organization, Geneva, Switzerland; 3 Consultant, New York, NY, USA; 4 Sigma 3 Services SÀRL, Nyon, Switzerland

**Keywords:** human rights, sexual and reproductive health, HIV, prevention, pre-exposure prophylaxis, microbicide

## Abstract

**Introduction:**

Two new microbicide products based on topical (vaginal) application of antiretroviral drugs – 1% tenofovir gel and the dapivirine ring – are currently in late-stage clinical testing, and results on their safety and effectiveness are expected to become available in early 2015. WHO guidelines on the use of topical pre-exposure prophylaxis (topical PrEP) are important in order to ensure that these new prevention products are optimally used.

**Discussion:**

Given that these new topical PrEP products are designed to be woman initiated and will likely be delivered in reproductive health settings, it is important to ensure that the guidance be framed in the context of comprehensive sexual and reproductive health and human rights. In addition to the safety and effectiveness data resulting from clinical trials, and the regulatory approval required for new products, the WHO normative guidelines on the use of topical PrEP will be essential for rapid roll-out in countries.

**Conclusions:**

Human rights standards and principles provide a framework for the provision of woman-initiated HIV prevention products. These include addressing issues related to the gender inequities which are linked to the provision of HIV-prevention, treatment and care for young girls and women. Effective programming for women and girls must therefore be based on understanding the local, social and community contexts of the AIDS epidemic in the country, and adapting HIV strategies and programmes accordingly. Such a framework therefore is needed not only to ensure optimal uptake of these new products by women and girls but also to address sociocultural barriers to women's and girls’ access to these products.

## Introduction

Two new microbicide products based on topical (vaginal) application of antiretroviral drugs for HIV prevention – 1% tenofovir gel and the dapivirine intravaginal ring – are currently in late-stage clinical testing and results on their safety and efficacy are expected to become available in early 2015.

To ensure that these products are able to reach women and young girls most at risk of HIV acquisition, their launch must be accompanied by WHO guidelines on the use of topical pre-exposure prophylaxis (topical PrEP). These guidelines must be evidence-based and integrate human rights standards and principles with recommendations taking into account policy, programmatic and community considerations. These guidelines are important to ensure that new topical PrEP products are implemented in high HIV incidence countries and settings in a manner that is equitable and effective. WHO recommendations have a strong influence on policy, can accelerate implementation in resource-limited settings and are necessary for accessing funding from some donors. They contain clinical, public health and policy recommendations about health interventions, and provide information about what policy makers, health-care providers or patients should do [1]. WHO has adopted internationally recognized standards and methods for guideline development [2] to ensure that guidelines are unbiased and that the products or interventions meet a public health need. The recommendations are based on a comprehensive and objective assessment of the available evidence and the process used to develop the recommendations is clearly described. WHO guidelines are complementary to regulatory review and approval. While national regulatory authorities have the responsibility to determine whether a product should be allowed onto the market in their jurisdiction, WHO guidelines provide advice for national programme managers and policy makers as they decide whether and how a product should be used. This advice addresses, for example, whether and how it should be prioritized to certain segments of the population or risk groups, and how to deliver the new product in an efficient and cost-effective manner with due consideration to other priority health interventions.

Given that new topical PrEP products are designed to be initiated by the woman herself and will likely be primarily delivered in reproductive health settings, it is important to ensure that national programmes are designed and services delivered within a framework of comprehensive sexual and reproductive health and human rights. Human rights standards, principles and treaties provide guarantees specifically relating to access to contraceptives. In addition, they recommend, among other actions, that states should ensure timely and affordable access to good quality sexual and reproductive health information and services, including contraception, which should be delivered in a way that ensures fully informed decision-making, respects dignity, autonomy, privacy and confidentiality, and is sensitive to individuals’ needs and perspectives. These guarantees are essential prerequisites to ensure women and girls are able to access and use the new HIV prevention methods.

WHO has recently issued guidance on ensuring that human rights are integrated into the provision of contraceptive information and services with a total of 24 recommendations on how this can be achieved [3]. Promotion and protection of human rights of women and girls are at the centre of this approach, including their right to have control over and decide freely and responsibly on matters related to their sexual and reproductive health, free of coercion, discrimination and violence. This requires governments to adopt and accelerate the implementation of laws, policies and programmes which protect and enable the enjoyment of all human rights and fundamental freedoms, including their reproductive rights in accordance with the International Conference on Population and Development (ICPD) Programme of Action [4], the Beijing Declaration and Platform for Action [5] and the 2004 WHO Global Reproductive Health Strategy [6]. Human rights are guaranteed in international and regional treaties, as well as in national constitutions and laws. They include the right to non-discrimination; the right to life, survival and development; the right to the highest attainable standard of health; and the rights to education and to information.

These rights have been applied by international, regional and national authoritative human rights bodies – such as UN treaty-monitoring bodies, international and regional courts, and national courts – to a wide range of sexual and reproductive health issues, including HIV, sexually transmitted infections (STIs), and recently to the accessibility of contraceptive information and services. This approach also resonates with global health programmes such as the PEPFAR Gender Strategy which recognizes that gender inequalities increase women's and girls’ vulnerability to HIV and must be addressed in designing and implementing HIV programmes, in particular the HIV prevention programmes [7]. The investment to develop new woman-initiated methods for HIV prevention has been driven by the recognition that there remain important gaps in HIV prevention, particularly for young women in generalized HIV epidemic settings, who remain at high risk of infection, even in clinical trial settings with intensive HIV risk reduction interventions. This is well illustrated by the HIV incidence among women in the placebo arms of recent randomized controlled trials of novel HIV prevention methods – 5.0 per 100 person years in the FEM-PrEP trial of oral PrEP trial conducted in Kenya, South Africa and Tanzania [8], 5.7 in the VOICE trial of oral and vaginal products conducted in South Africa and Uganda [9], 5.9 in the Phambili vaccine trial in South Africa [10] and 9.1 in the CAPRISA 004 trial of 1% tenofovir gel [11]. While condoms are highly effective in reducing the risk of HIV infection in serodiscordant couples [12] and among sex workers [13], their use and impact remains stubbornly low in sex between regular partners [14, 15].

## Discussion

A significant challenge to designing and implementing programmes to deliver new HIV prevention methods is to ensure that they are made available to, and used by, women at high risk of HIV infection who are not using existing methods. Many such women visit family planning and other reproductive health services to obtain contraceptive advice and supplies, or to seek treatment for STIs. Thus, these family planning and reproductive health services have a key role to play in providing information on the new HIV prevention methods, even if a woman can only obtain the products from a limited number of facilities. Making the products available to women who do not access family planning or other reproductive health services will be a greater challenge and innovative programmatic approaches must be developed.

Building upon existing human rights standards and principles, several issues are particularly relevant in the context of provision of woman-initiated HIV prevention products. These include, but are not limited to, improving quality of services through:

1. integration of and/or linkages between reproductive health and HIV services which provide topical HIV prevention, condoms and contraceptive commodities, supplies and equipment, covering a range of methods, including emergency contraception.
2. providing evidence-based, comprehensive information, education and counselling to ensure informed choice with regard to contraception, and to prevention and care for HIV and other STIs.

The ICPD Programme of Action recognizes that women and adolescent girls, especially those who live in impoverished and otherwise disadvantaged communities and circumstances, disproportionately bear the greatest costs and consequences of failure to promote and protect sexual and reproductive health and reproductive rights. However, many women who would benefit most from these new HIV prevention products are young women and adolescents who, in many communities, have poor access to services. They may live in contexts where health-care providers do not have the training or the means to provide young people with age-appropriate services, or choose not to do so based on their own biases. Legal and policy barriers, such as parental consent requirements, can also inhibit girls’ access to services. Issues of confidentiality, women's empowerment and participation and accountability are essential to ensure promotion and protection of sexual and reproductive health and human rights of women and girls. For instance, women's privacy, including confidentiality of medical and other personal information, needs to be respected throughout the provision of HIV and STI prevention and care, and contraceptive information and services. For some women, using, or trying to use, one of the new HIV prevention products may expose them to violence, or exacerbate the risk of intimate partner violence. The ICPD Programme of Action also obliges governments to ensure availability of and access to the information, comprehensive sexuality education, and quality sexual and reproductive health services necessary to ensure sexual and reproductive health and enjoy human rights.

Results from the CAPRISA 004 trial announced in 2010 showed that 1% tenofovir gel used around the time of intercourse reduced the risk of HIV infection by 39% among women in KwaZulu-Natal [11] and the FACTS001 study of the same product, currently underway in eight sites in South Africa, is expected to announce preliminary results in early 2015 [16]. This is in contrast to the VOICE trial of daily gel use which showed no reduction in HIV incidence when users were instructed to use the product every day irrespective of anticipated or actual sexual intercourse [9]. The failure to show any protective effect was attributed to poor adherence to daily product use. The product in a coitally dependent regimen is not likely to be licensed for use for at least two years, given the time necessary to collate data, prepare the regulatory dossier, submit to national regulatory authorities and allow in-depth regulatory review. It will be a further one or two years before the product becomes more widely available to women at high risk of HIV infection who choose to use it. A similar process and timeframe is expected for the second topical microbicide product in development, a vaginal ring containing the antiretroviral dapivirine which is currently in two parallel Phase 3 trials in Malawi, South Africa, Uganda and Zimbabwe [17, 18]. The intervening time from the end of Phase 3 trials to product availability provides an opportunity to implement operational research among former trial participants, new users and/or communities that have not been previously involved in the research. A carefully designed programme of operational research will inform service delivery approaches to best make the products available and support women in their use.

In contrast, there was no opportunity to implement operational research and learn how to design access programmes before oral PrEP was licensed in the United States in 2012. In 2010, the iPrEx study showed that daily oral tenofovir disoproxil fumarate and emtricitabine (TDF/FTC) reduced the risk of HIV infection by 44% among men and transgender women who have sex with men [19]. Additional studies published in 2012 showed that TDF alone or TDF/FTC was also effective in reducing HIV infection in heterosexual men and women in known serodiscordant couples, injecting drug users and other high HIV risk men and women [20–22]. The combination TDF/FTC was approved for use in HIV prevention by the US Food and Drug Administration and WHO released programme guidance in July 2012 with a provisional recommendation on use within the context of demonstration projects for serodiscordant couples, and men and transgender women who have sex with men [23]. The absence of programmatic experience or implementation research on how to deliver oral PrEP to users prevented more specific recommendations from being made at that time. A wide array of introductory and demonstration projects on oral PrEP are now being developed and implemented. These demonstration projects will provide important information over the next two years on acceptable and sustainable programme design, how to reach men and women at risk of HIV infection who wish to access and use oral PrEP and how to retain them in a comprehensive combination HIV prevention programme. However, the majority of the ongoing or planned demonstration projects are in serodiscordant couples, sex workers and men or transgender women who have sex with men. Only 2 of the 26 oral PrEP demonstration projects listed by AVAC in December 2013 involve women at risk of HIV infection who are neither sex workers nor in a stable, known serodiscordant relationship [24]. It is these women at risk of HIV infection who are likely to pioneer use of, and benefit most from, topical PrEP.

In March 2014, WHO and CAPRISA convened a stakeholder consultation to identify priority implementation research on tenofovir gel and dapivirine ring to inform development of WHO guidelines on the use of topical PrEP [25]. The consultation brought together a range of stakeholders to determine what issues were most critical for WHO guideline development, and the research approaches and timing where they can be addressed. Building on research questions and information gaps identified at previous consultations convened since the release of the CAPRISA 004 results, consultation participants prioritized key operational and policy research priorities for the provision of topical PrEP (Table 1).

**Table 1:** Key operational and policy research priorities for provision of topical PrEP

Priority area	Research elements
**Define the core service package** to deliver tenofovir gel and dapivirine ring safely and appropriately in different service delivery settings (family planning clinics, HIV testing sites, sexual and reproductive health services)	• HIV testing and retesting models (including frequency, location and self-testing or provider-led testing) • Prescribing and resupply models (location of initial supply and refills, frequency of product dispensing) • Counselling approaches • Whether and how to monitor for drug resistance in newly infected product users • Frequency and methods for measuring adherence • Barriers to use (travel, time in clinic)
**Determine how best to provide topical PrEP to adolescents and young women** (aged 15 to 24)	• Clinical safety studies in women below age 18 years to allow labelling for use in this age group • Feasibility and acceptability of delivering tenofovir gel and dapivirine rings to young women • Willingness and ability to use the product • How to offer topical PrEP without undermining condom use • Young women's beliefs about the products, risks and how these might affect adherence • Site-specific situation analyses to identify where young women go, or would go, to access topical PrEP for HIV prevention
**Support consistent product use**	• Tools to determine which factors influence use, and identify reasons for non-use • Examine factors at multiple levels that influence product use, and can be complemented by objective measures of adherence • Develop approaches that allow support to be tailored to the individual user • Ongoing assessment of adherence to products known to be safe and effective
**Develop provider training materials**	• Develop, test and adapt materials as experience in implementing programmes and supporting users accumulate • Draw on clinical trial documents and evidence as well as available training materials from other sexual and reproductive health and HIV prevention technologies • Practical tools for screening potential users to be used by providers and women to help identify those at high risk of HIV, those likely to adhere to product use, and other approaches for defining users
**Conduct policy analysis and synthesis to build an enabling environment**	• Synthesize evidence, outstanding questions and approaches to monitoring and minimizing resistance to address policymaker concerns • Synthesize evidence, outstanding questions and approaches to monitoring and minimizing risk compensation to address policymaker concerns • Identify the best strategies and approaches to demand creation • Determine how best to anticipate and address social issues (partner testing and disclosure, preventing and addressing intimate partner violence, considerations for sex workers) • Identify needed data to inform consistent approach and parameters for modeling cost effectiveness, impact and other key outcomes

In addition, stakeholders at the consultation noted that programme implementation of tenofovir gel and dapivirine ring could be addressed in each of the three phases of research: 1) open label extensions of the ongoing clinical trials; 2) pilot introductory studies and operational research after confirmation of safety and efficacy, but before product licensure; and 3) programmatic research following product licensure (Table 2). The stakeholder consultation strongly reaffirmed that implementation research and roll-out should be prioritized in communities and countries that have hosted clinical trials of topical PrEP and other HIV prevention trials in women, including full support for open label extension studies in the sites where trials of tenofovir gel and dapivirine ring are ongoing.

**Table 2:** Research phases and characteristics of operations research studies

Phase of research	Settings/key characteristics	Key outcomes
• Before product licensure Open label extension of efficacy trials	Efficacy trial sites Participation limited to efficacy trial participants All are former trial participants and successful prior users	Additional safety data to support licensure Key questions to allow less restrictive labeling: e.g. • Frequency of HIV testing, product resupply • Adherence support models • Alternative service delivery settings
Pilot introductory studies and operations research (following safety and effectiveness, prior to licensure)	New users Can be in trial sites or other settings Research requires ethical and regulatory approvals and individual informed consent	Inform guidance and product labeling Inform initial programme design (e.g. minimum service package, efficient adherence support models, feasible and sustainable service delivery models) Extend to new user groups (e.g. extended age range, relaxed eligibility criteria and extension to users previously excluded due to minor contraindications)
• After product licensure Demonstration projects/implementation research	New users Projects developed to inform programme design Research designed and conducted in the context of ongoing programmes	Inform successful programme scale up and adaptation Inform development of updated guidance as experience with product delivery and use accumulates Address public health use of products

## Conclusions

Given the continued high rate of new infections among women, especially young women in generalized HIV epidemics, it is critical to determine how best to ensure that effective HIV prevention products, including topical PrEP, can be delivered appropriately and sustainably. Given the gender inequities that drive the need for women-initiated products, WHO guidance on topical PrEP must not only build upon relevant existing WHO guidelines but also be grounded in a comprehensive sexual and reproductive health and human rights framework. Such guidance will be highly influential in determining how these products are provided, accelerating programme uptake of new technologies in resource-limited settings, and facilitating purchase and programme support by international agencies and donors.

Timely design and implementation of a programme of introductory and demonstration studies on topical PrEP will ensure that the period between confirmation of safety and efficacy and product licensure is best used. This period can generate new knowledge on how to deliver the products to users in an effective and sustainable manner, how to retain users in prevention programmes, what mix of HIV prevention methods can be effectively and efficiently delivered together, how to integrate with existing sexual and reproductive health programmes and how to involve the community and potential users in programme design, implementation and monitoring.
